# Palliative Radiation Therapy for Symptom Control in an Advanced Case of Pseudomyxoma Peritonei

**DOI:** 10.7759/cureus.1407

**Published:** 2017-06-29

**Authors:** Clare McGrath, Kelly Linden, Pamela Hube, Anna Adamiak, Kristopher Dennis

**Affiliations:** 1 Faculty of Science, Biomedical Science, The University of Ottawa; 2 Department of Radiation Medicine, The Ottawa Hospital Cancer Centre; 3 Department of Pathology and Laboratory Medicine, The Ottawa Hospital; 4 Division of Radiation Oncology, The University of Ottawa

**Keywords:** palliative care, pseudomyxoma peritonei, radiation therapy, radiotherapy, supportive care, symptom control

## Abstract

Pseudomyxoma peritonei (PP) is a rare clinical condition characterized by progressive mucinous ascites, which is typically caused by a mucin-producing neoplasm. Reports of radiation therapy (RT) in the management of PP are limited. We report a unique case of a 62-year-old woman with severe, end-stage, recurrent PP and a large, mucin-secreting mass protruding through her abdominal wall. Low-dose, hypofractionated palliative RT was administered for symptom control with the hope of improving her quality of life. We suggest that radiation therapy be considered in the comprehensive palliative management of patients with PP.

## Introduction

Pseudomyxoma peritonei (PP) is a rare clinical condition of progressive mucinous ascites, which is typically caused by a mucin-producing neoplasm [1-2]. There is controversy regarding the classification of PP, and an ongoing debate regarding the origin, pathology, definition, and ideal classification of PP subtypes [1-2]. For example, the World Health Organization (WHO) classifies PP as either low grade or high grade. Ronnet et al. classify PP as disseminated peritoneal adenomucinosis (a more favorable low-grade process), peritoneal mucinous carcinomatosis (a less favorable high-grade process), or an intermediate-grade process between these subtypes [3]. The underlying neoplasm, often of appendiceal origin, can be a low-grade appendiceal mucinous neoplasm (LAMN) or a mucinous appendiceal adenocarcinoma [1,4].

Regardless of the PP subtype, its natural history is one of continuous peritoneal accumulation of mucin, which ultimately results in fatal multifocal bowel obstruction.

A common treatment for PP in operable patients, regardless of the subtype, is the resection of all gross disease, often via the cytoreductive technique described by Sugarbaker [5] or, at the minimum, extensive debulking surgery when all disease cannot be safely excised. Cytoreductive surgery is often combined with hyperthermic intraperitoneal chemotherapy (HIPEC) [2]. The rarity of PP and the lack of other mature data describing alternative therapeutic approaches could explain why cytoreductive surgery and HIPEC are still standard treatments, despite the high rate of major complications with this approach [2]. For patients deemed inappropriate for surgery, chemotherapy alone is rarely considered, but the few results that exist have been discouraging and no consensus exists on the ideal treatment for patients in this setting [6]. 

Reports of radiation therapy (RT) in the management of PP are limited [6-8]. Aggressive approaches using whole abdominal RT have been published in case reports or in small series, with mixed results [6-8]. However, the optimal role of RT in the management of patients with PP is unclear.

To add to the limited literature describing palliative RT for PP, we present a unique case of a patient with severe, end-stage, recurrent PP and a large, mucin-secreting mass protruding through her abdominal wall. Low-dose, hypofractionated palliative RT was administered for symptom control with the hope of improving her quality of life.

## Case presentation

A previously healthy 54-year-old woman presented with several years of progressive abdominal bloating. An ultrasound and a computed tomography (CT) scan revealed multiloculated bilateral adnexal cystic masses indistinguishable from the ovaries and measuring up to 11 x 15 x 17 cm. She underwent surgery for diagnostic and therapeutic purposes where intraoperatively, thick mucinous material was seen filling the abdominal cavity. Mucinous implants were seen along the right hemidiaphragm, the serosal aspect of the entire small bowel and large bowel, and throughout the pelvis. A hard nodule was encountered, involving the appendix, and the overall appearance seemed consistent with an appendiceal primary neoplasm with ovarian implants. Total abdominal hysterectomy, appendectomy, bilateral salpingo-oophorectomy, omentectomy, and right hemicolectomy were carried out. The majority of visible disease was resected, but mucinous implants across the right hemidiaphragm and the small and large bowels remained. Pathological examination confirmed a LAMN of the appendix with a focus on the rupture associated with pseudomyxoma peritonei. The hard appendiceal nodule encountered intraoperatively was an incidental, well-differentiated neuroendocrine carcinoma. Abundant mucin pools were implanted across all submitted specimens, with a few entrapped mucinous epithelial cells with low-grade atypia seen within. No primary malignancies were identified within gynecologic viscera.

Four weeks after surgery and initial diagnosis, mucin began leaking from the laparotomy incision, which had never completely healed. Four months after diagnosis, a CT scan revealed the progression of residual peritoneal carcinomatosis and the development of a new lesion along the hemidiaphragm and midline laparotomy site. The patient then underwent cytoreductive surgery with mitomycin C heated intraperitoneal chemotherapy (HIPEC). During the following months, her PP unfortunately progressed slowly, with periodic scans noting an increasing number of mucin deposits throughout the peritoneum. A number of these deposits were removed in a debulking surgery 62 months after the initial diagnosis, in an attempt to slow progression. Nevertheless, her disease progressed, most prominently in the right pelvis, with an enlarging mass extending into the musculature of the pelvic wall. Another debulking surgery was undertaken to address this.

Shortly after, the patient noticed a bulging mass in her right posterior flank and had pain radiating down her right leg. CT revealed a large peritoneal mass extending posteriorly to involve the right psoas and flank musculature. At 91 months after the initial diagnosis, the mass broke through the skin of her back and began oozing green mucin and giving off an odor. 

**Figure 1:**
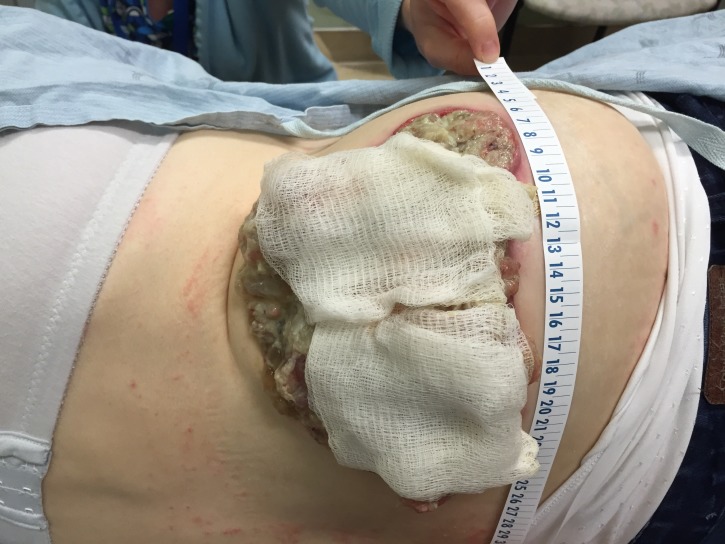
Protruding mucin-weeping mass, right posterior flank

**Figure 2:**
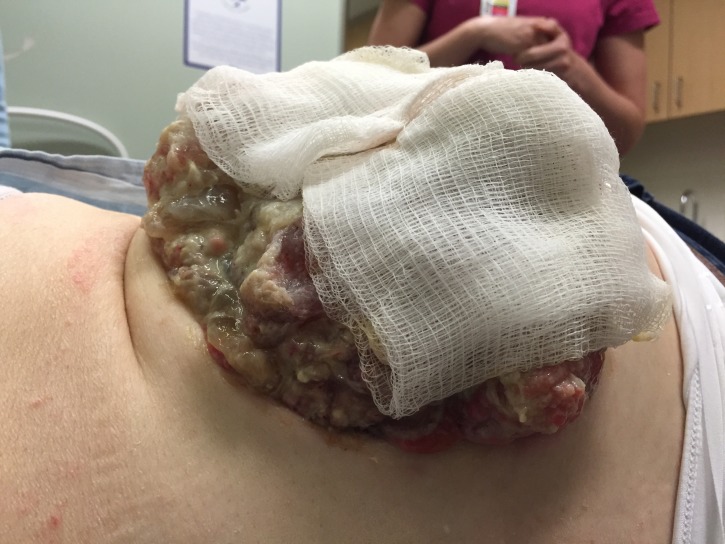
Bandages soaked with mucin

**Figure 3:**
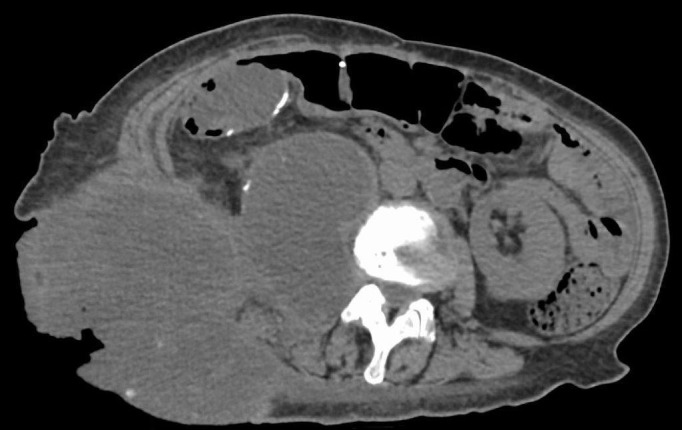
Unenhanced axial CT image showing confluent intra- and extra-abdominal disease

At 95 months from diagnosis, the patient was referred to radiation oncology to address the protruding right flank mass that had grown to the point where she could not lie supine. She had pain locally as well as down the right leg when walking because of the psoas muscle infiltration. Mucin was oozing in quantities that required several sessions daily, where she would wash off the excess with a shower head and apply new absorbent dressings, which were becoming expensive. Nausea was also bothersome because of slowed gastrointestinal motility and opioids. Her Eastern Cooperative Oncology Group (ECOG) performance status was three. On examination, the visible component of the mass measured approximately 24 x 14 cm at its base and extended between 7 and 9 cm from the skin surface (Figures 1-2). A CT measured the confluent intra-abdominal component of the mass at 13 x 12 x 9.5 cm (Figure 3). Numerous other deposits and mucin filled the rest of the abdominopelvic cavity.

**Figure 4:**
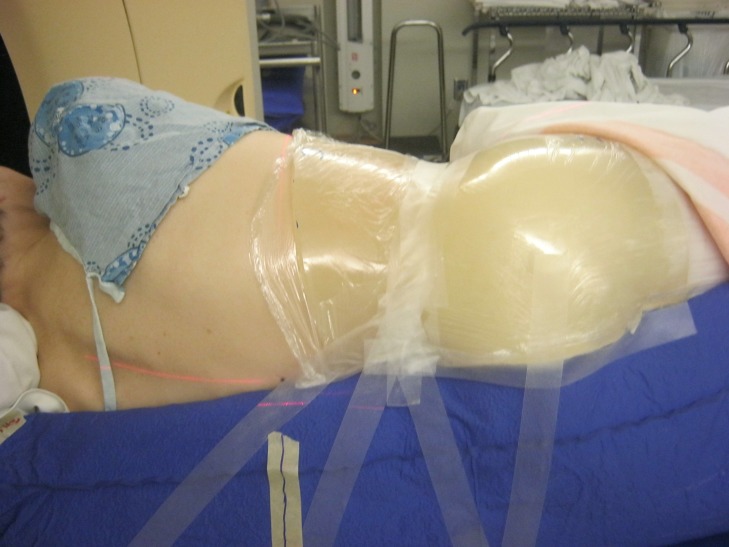
Radiation therapy simulation set-up with patient lying on her uninvolved side within a full-body vac-lock bag with a 1 cm bolus over bandaged, protruding mass

Given her poor performance status, pain, and extent of disease, we administered a palliative dose of 16 Gy in two fractions, with fractions spaced one week apart and a primary goal of reducing the volume of mucin oozing from the mass to improve tumor hygiene, decrease the frequency of dressing changes, and improve her quality of life. Pain relief was a secondary goal. She underwent CT simulation lying on her uninvolved side, supported by a full-body vac-lock bag for immobilization. One cm of tissue-equivalent bolus was applied over the bandaged protruding mass [Figure 4]. Treatment was administered with 6 MV photons via volumetric modulated arc therapy (VMAT) to minimize GI toxicity. The gross tumor volume (GTV) included all disease seen on imaging and clinical examination. The clinical target volume (CTV) was a 1-cm isotropic expansion from the GTV, excluding uninvolved muscle, viscera, and bone. The planning target volume (PTV) was a 1-cm isotropic expansion from the CTV. Cone beam imaging was used to verify patient positioning. She was administered an 8 mg orally disintegrating tablet of ondansetron before each fraction for radiation therapy-induced nausea and vomiting (RINV) prophylaxis and instructed to take the same medication for two days after each fraction. She was given 2 mg of hydromorphone subcutaneously for incident pain moving on and off the treatment couch. She tolerated treatment relatively well despite her incident pain.

**Figure 5:**
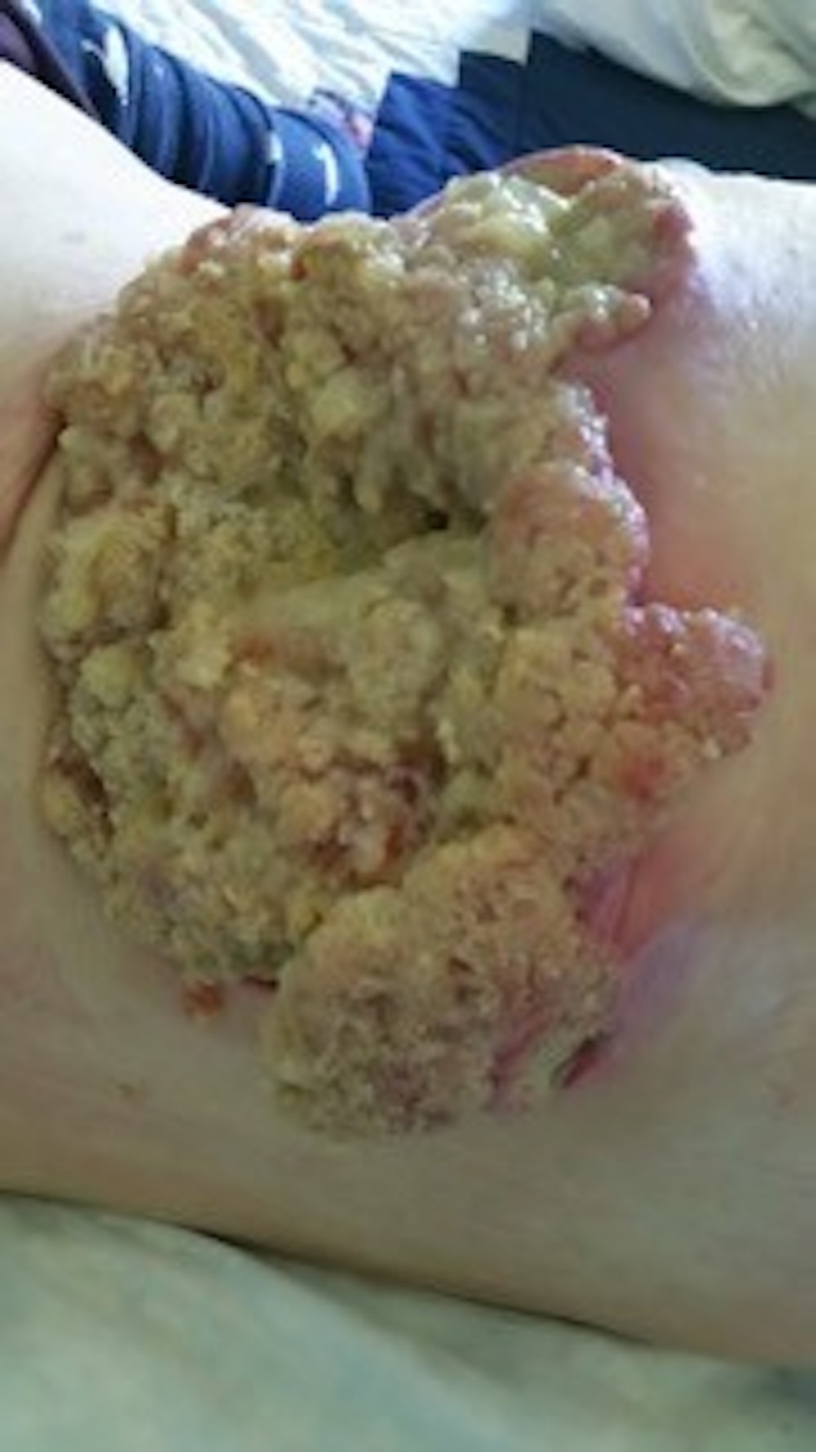
Protruding right flank mass eight weeks following radiation therapy with significantly reduced mucin output

Within two weeks of her first fraction, she reported a more dried-out appearance of the protruding mass and the frequency of the dressing changes had decreased significantly [Figure 5]. Pain in that location had also improved. During follow-up, she did not report having noticed a worsening in her nausea after both radiation treatments. Unfortunately, in the following weeks, her generalized abdominal pain progressed, her performance status deteriorated, and she passed away in hospice at three months following her last fraction, approximately eight years from the time of her initial diagnosis. 

## Discussion

This is the first report of which we are aware that describes a gross disease caused by PP protruding through the skin to such an extent. In addition to the problems caused by her intra-abdominal and pelvic disease, our patient was significantly burdened by her protruding, weeping mass and in need of a comprehensive plan for symptom control. 

Low-grade PP [1] and disseminated peritoneal adenomucinosis [3] are considered to be more indolent PP subtypes, with better prognoses than high-grade PP [1] or peritoneal mucinous carcinomatosis [3]. Our patient’s overall pathological features and clinical behavior seemed to place her disease somewhere in between these entities. No frankly invasive adenocarcinoma or high-grade atypia was identified pathologically, but her disease spread and recurred aggressively.

Ronnett et al. showed that when the same surgeon treated patients with PP-related tumors uniformly, the five-year overall survival was 75% for indolent PP, but only 14% for malignant PP [3]. Because prolonged survival seems linked to a complete surgical resection of all visible disease [9], the deposits that could not be resected from our patient during her first surgery likely contributed to the aggressive natural history of her disease.

Whole abdominal radiation therapy has been used in advanced cases of PP to reduce the risk of progression following debulking surgery [6-7], and to treat symptomatic bowel obstruction [8]. Localized radiation therapy has been used to treat more isolated, yet symptomatic, areas of progression [7]. Similar to the "drying" effect that radiation therapy has on various symptomatic tumors, it is speculated that a component of the response of PP to radiation therapy might be from a decrease in mucin secretion. It was this hypothesis that motivated us to treat the weeping protruding mass.

Whole abdominal, abdominopelvic, and strip abdominal radiation therapy for PP have traditionally been delivered via simple anterior/posterior opposed beam arrangements (AP/PA). However, significant gastrointestinal toxicities have surprisingly not been reported [7] and under-dosing portions of the target volume because of a need to shield sensitive structures, such as the kidneys, potentially limit the effectiveness of these techniques. Newer, highly conformal techniques, such as volumetric modulated arc therapy (VMAT), have recently shown improved target coverage and the sparing of organs at risk when used for whole abdominal therapy [10]. We deemed VMAT the best option for our patient out of a desire to control the dose to the kidneys, liver, and spinal canal and to shield as much uninvolved small bowel as possible to decrease acute toxicity. Because she was already experiencing nausea due to opioids and slowed motility, minimizing RINV by controlling the dose to the small bowel was a major planning priority. As she did not report a worsening of her baseline nausea after treatment, our hope is that our strategy of highly conformal planning and RINV prophylaxis contributed to good iatrogenic symptom control.

## Conclusions

In summary, we present a unique case report that contributes to the limited literature describing radiation therapy in the management of patients with PP. Our patient’s protruding tumor had a favorable response to hypofractionated palliative RT, with a significant reduction in weeping mucin, which decreased the need for repeated washings and dressing changes. We suggest that RT be considered in the comprehensive palliative management of patients with PP.
